# Optical coherence tomography angiography in diabetic retinopathy: a review of current applications

**DOI:** 10.1186/s40662-019-0160-3

**Published:** 2019-11-18

**Authors:** Kai Yuan Tey, Kelvin Teo, Anna C. S. Tan, Kavya Devarajan, Bingyao Tan, Jacqueline Tan, Leopold Schmetterer, Marcus Ang

**Affiliations:** 1 Hobart Clinical School, Level 3, 43 Collins Street, Hobart, TAS 7000 Australia; 2 Singapore National Eye Centre, 11 Third Hospital Ave, Singapore, 168751 Singapore; 3 Singapore Eye Research Institute, 20 College Road Discovery Tower, Level 6 The Academia, Singapore, 169856 Singapore; 4 Singapore National Eye Centre, 11 Third Hospital Ave, Singapore 168751; Duke-NUS Medical School, 8 College Rd, Singapore, 169857 Singapore

**Keywords:** Optical coherence tomography angiography, Fluorescein angiography, Diabetic retinopathy, Screening, Monitoring

## Abstract

**Background:**

Diabetic retinopathy (DR) is a leading cause of vision loss in adults. Currently, the standard imaging technique to monitor and prognosticate DR and diabetic maculopathy is dye-based angiography. With the introduction of optical coherence tomography angiography (OCTA), it may serve as a potential rapid, non-invasive imaging modality as an adjunct.

**Main text:**

Recent studies on the role of OCTA in DR include the use of vascular parameters e.g., vessel density, intercapillary spacing, vessel diameter index, length of vessels based on skeletonised OCTA, the total length of vessels, vascular architecture and area of the foveal avascular zone. These quantitative measures may be able to detect changes with the severity and progress of DR for clinical research. OCTA may also serve as a non-invasive imaging method to detect diabetic macula ischemia, which may help predict visual prognosis. However, there are many limitations of OCTA in DR, such as difficulty in segmentation between superficial and deep capillary plexus; and its use in diabetic macula edema where the presence of cystic spaces may affect image results. Future applications of OCTA in the anterior segment include detection of anterior segment ischemia and iris neovascularisation associated with proliferative DR and risk of neovascular glaucoma.

**Conclusion:**

OCTA may potentially serve as a useful non-invasive imaging tool in the diagnosis and monitoring of diabetic retinopathy and maculopathy in the future. Future studies may demonstrate how quantitative OCTA measures may have a role in detecting early retinal changes in patients with diabetes.

## Background

Diabetes is currently on the rise with 422 million of people in the world reported to have diabetes in 2014 [1] and is a systemic disease with a multitude of complications which may involve the eyes. The most common ocular complication is diabetic retinopathy (DR), which may be asymptomatic in the early stages, however, disease progression can lead to severe vision loss [2]. Diabetic retinopathy is a leading cause of blindness in working age adults [3] and is estimated to affect 1 in 3 diabetic patients [4, 5]. Diagnosis of DR is based on clinical findings and can be divided into 2 categories - early non-proliferative diabetic retinopathy (NPDR) and more advanced proliferative diabetic retinopathy (PDR) associated with retinal ischemia and development of neovascularisation [6]. The main sight-threatening complications of DR are diabetic maculopathy, which include diabetic macular oedema (DME) and diabetic macular ischemia (DMI) [7], and complications from PDR - vitreous haemorrhage and retinal detachment [8]. Digital retinal fundus image analysis has been shown to be able to detect early DR and DME in routine DR screening [9–11]. While it has high sensitivity and specificity, it has been shown to have a low negative predictive value [11].

**Fig. 1:**
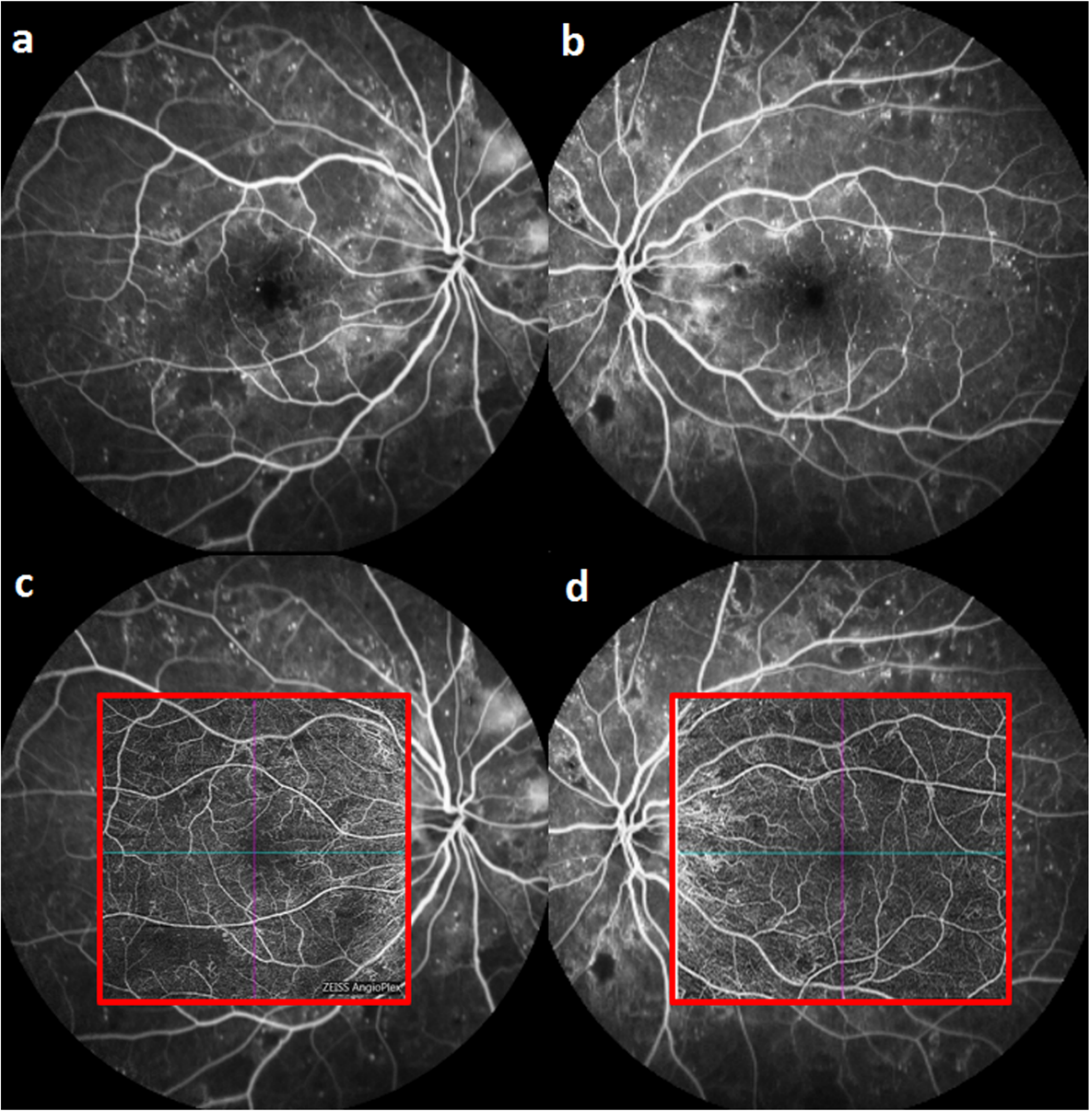
Comparison of Fluorescein Angiography and OCTA. **a** & **b** Fluorescein angiography images of a patient with proliferative diabetic retinopathy. These FA images show patchy areas of capillary drop out and presence of neovascularizations elsewhere (NVE). **c** & **d** Corresponding OCTA images (generated via ZEISS AngioFlex) of (**a**) and (**b**) being superimposed on the FA images. The OCTA images also show areas of capillary drop out and new vessels without leakage

Optical Coherence Tomography (OCT) offers a non-invasive, rapid imaging modality that can provide imaging of the cross-sectional structures of the retina by using low-coherence interferometry to capture high resolution two dimensional images from the optical scattering from different layers of the retina [12] and is an essential tool in the detection and monitoring of DME [13], and DMI with inner retinal thinning [14]. Optical coherence tomography angiography (OCTA) is a novel use of OCT to visualise the microvasculature of the retina and choroid without the need for dye injection [15]. This is performed through repeated scans at the same location to detect the changes in OCT reflectance signal from the flow through blood vessels [16, 17]. It allows depth-resolved imaging of the retinal vasculature and is an ideal approach for various retinal conditions such as DR, retinal venous occlusion, uveitis, retinal arterial occlusion and age-related macular degeneration [18, 19].

In this review, we will discuss the role of OCTA in the evaluation and monitoring of DR, diabetic maculopathy and the anterior segment involvement in DR.

## Main text

### Literature search

We conducted a literature search via PUBMED database for articles written in the English language until January 1, 2019, with the following medical subject headings: “OCTA,” “OCT angiography,” “Diabetic Retinopathy,” or “Diabetes”. All papers that used OCTA were reviewed for findings in DR and bibliographies were hand-searched for further studies. Eighty-eight articles were identified, with 11 papers being excluded as they were either reviews, inter-instrumental reliability study or case report/series. There was a total of 58 prospective studies, of which 17 were observational, 30 were observational and cross-sectional, and 11 were observational case-control study. There was a total of 19 retrospective studies, of which 12 were observational, two were observational cross-sectional, two were case-control and three were cross-sectional. In total, there were two multi-centred studies. The number of patients vary widely among studies. In addition to that, we also performed an additional search via PUBMED database with the following medical subject headings: “OCTA”, “Anterior Segment”, which returned 27 articles, of which three articles were excluded as they were either reviews or case-report.

### Fluoresceine angiography and optical coherence tomography

Fluorescein angiography (FA) is helpful in the evaluation of the retinal vasculature and was first described in 1961 and later adopted as a standard practice in the field of Ophthalmology [20]. Fluorescein angiography can be used to evaluate the retinal vasculature to monitor the progression of DR and DME [21, 22]. In FA, sodium fluorescein is injected intravenously and with the use of excitation and barrier filters, high contrast en face images of the retinal vasculature can be visualised [23]. The advantage of FA lies in its ability to assess properties such as perfusion (e.g., arm-retinal time, arterio-venous transit), leakage and staining [24]. Flash photography and recently, scanning laser ophthalmoscopy can be used to capture FA images to allow visualisation of the retinal vessels in high contrast [25, 26]. With ultra-widefield FA, the imaging field can visualise the entire posterior segment and extend beyond the equator of the eye, giving a field of view of up to 200 degrees [27].

FA is a primary en face modality, and cross-sectional segmentation of the retinal vessels is not possible [28]. Depth resolution is inferred from FA, and indocyanine green angiography (ICGA) can be used to differentiate choroidal from retinal perfusion as it has a larger molecule size [28].

On the other hand, OCTA has several advantages over dye angiography in terms of acquisition speed and imaging information (Fig. 1) [28]. OCTA images are essentially motion-contrast images with images obtained via multiple B scans at the same location, and information derived is based on the backscattering of light from the changes in the intensity and phase from each scan changes due to blood flow while the neurosensory tissue will remain stationary, henceforth this approach eliminates the need for dye. The primary advantage of OCTA is the ability to obtain depth-resolved imaging of the retinal vasculature [29]. It is able to generate the images of the superficial and deep retinal layers by default [30] and this can be modified to further segment the retinal vasculature and provide images of other layers such as the radial peripapillary network and choriocapillaris [28, 31, 32], which can help to visualise pathological features that are not previously seen in 2-layers segmentation [32]. The corresponding flow signal on OCT B-scans allows cross-sectional localization of the vasculature in question.

There are several shortcomings for OCTA use. Firstly, the field of view of OCTA is narrower than FA, with most images being 3 mm by 3 mm [28]. The largest scanning area that is achievable with commercially available OCTA devices is 8 mm by 8 mm which grants a field of view of approximately 30 degrees [33]. Thus, OCTA has poor ability in generating good quality peripheral retinal images [28]. Even with the introduction of wide-field OCTA that is able to generate images of 12 mm by 12 mm the field of view is still not comparable to standard and ultra wide-field FA/ICGA [34, 35]. To overcome this limitation, the montaging algorithm has been introduced which allows the 12 mm by 12 mm images to be montaged and generate a wider field of view [28]. This approach, however, results in an increase of scan acquisition time, and inherent inaccuracies due to misalignment of images [36]. Secondly, OCTA is unable to assess dynamic characteristics of flow velocity, and leakage which is sometimes necessary to assess various retina pathology. Thirdly, processing of high-resolution images can be time-consuming [37] and images generated via OCTA are highly susceptible to projection artefacts due to the presence of the superficial blood flows resulting in difficulty in interpreting the deep retinal vasculatures [38]. While this can be corrected via projection removal algorithms, this method may potentially result in loss of flow information within the deeper layer, giving a disjointed image [39]. Additionally, OCTA images are prone to motion artefacts as well, which often appear as a white line across the image, and can be improved with motion correction function and eye-tracking algorithm [40].

### Morphological changes of DR on OCTA

Several morphological changes of DR can be detected by OCTA – microaneurysms (MAs), intraretinal microvascular abnormalities (IRMAs), nonperfusion areas and neovascularizations (NVs) [41], and it is able to offer additional information with respect to the localization of these changes [42].

Microaneurysms are lesions that often manifest in early DR. Thompson et al. showed that OCTA is able to pick up MAs, not otherwise shown on a dilated clinical examination [43]. OCTA is able to localize MAs precisely [42]. There are, however, discrepancies, among studies, in regards to the detectability of MAs between FA and OCTA [42, 44–46]. FA has demonstrated higher sensitivity compared to OCTA [47–49]. On the other hand, the majority of MAs detected by OCTA has a corresponding finding in FA [45]. Schwartz et al. and Ishibazawa et al. demonstrated that OCTA can detect MAs that are otherwise not detectable on FA [42, 46]. Detection of MAs using OCTA, however, may be influenced by blood flow turbulence within the MAs [50] and hence the discrepancy found among the studies [41, 48, 51, 52]. Parravano et al. have identified a correlation between the MAs’ reflectivity and its detectability on OCTA – MAs that are hyper-reflective are more likely to be detected but this may also be affected by turbulent blood flow in MAs [53]. As such, it is still unclear if OCTA is comparable to FA in terms of detecting MAs.

Intraretinal microvascular abnormalities are shunt vessels due to abnormal branching or dilation of existing capillaries within the retina that help to supply areas of non-perfusion in DR. Visualisation of IRMAs has been made possible with OCTA via the use of en face images and are shown as dilated or looping vessels near the areas of capillary loss, and has a higher detection rate on OCTA than color fundus photography [54]. The use of OCTA also allows identification of other features such as the presence of intraretinal hyperreflective dots and outpouching of the internal limiting membrane (ILM) [55], which may be useful in detection of IRMAs.

Retinal NVs are detectable on OCTA via observation of flow signal above the ILM [55]. OCTA can detect early retinal NVs [49] and identify the origins and morphological patterns of NVs in PDR, hence allowing classification of the lesion, offering a better understanding of the pathophysiology and helps to guide the management strategies [56]. OCTA is also able to detect subtle NVs, which is difficult to differentiate from a MAs on FA [49].

Owing to OCTA’s ability to segment the various layer of the retina, it is able to distinguish retinal NVs from IRMAs, which may not always be possible on FA or clinical examination [44], and is of importance as very often, retinal NVs may form next to IRMA [55]. In addition to that, de Carlo et al. showed that retinal NVs often appear next to retinal non-perfusion areas [55]. As such, OCTA may be useful in helping us to differentiate NPDR from DR, and aid us in following up and management planning.

### Quantitative measures in OCTA and its application in DR

Various quantitative measures have been developed over the years to aid research studies as well as the understanding of DR pathophysiology. These quantitative measurements have been shown to allow objective identification and staging of NPDR – mild, moderate and severe, with significant diagnostic accuracy and predictability of DR progression [57]. To the best of our knowledge, we are not aware of any normal data material available for the different OCTA measurements. Several OCTA vascular quantitative measures currently used in research and has yet to be adopted in clinical practice have been proposed:

1. The area filled by binarized vessels (vessel area density – VD or vessel perfusion density - PD) [57–60];
2. Vessel spacing/inter-capillary area [61];
3. Length of the blood vessel based on the skeletonized OCTA (vascular length density – VLD or skeleton density - SD) [57, 59];
4. Vessel diameter index (VDI) [57];
5. Total length of vessels (vessel length fraction) [62];
6. Vascular architecture and branching, (vessel tortuosity and fractal dimension – FD) [58];
7. Area of the foveal avascular zone – FAZ [63].

Certain commercially available devices – Topcon DRI-OCT Triton Swept-source OCT, Optovue RTVue-XR, Heidelberg-Engineering and Zeiss Cirrus 5000-HD-OCT enhance efficiency and reduce bias as they automatically map VD, FAZ and PD [64, 65]. In general, VD, SD, FD and VDI are highly reproducible among graders and studies have found that vascular changes in DR may be characterized by these parameters [66].

#### Vessel density

Vessel density is defined as the proportion of blood vessel area over the total measured area [67]. Measurements of VD are highly reproducible and comparisons of measurement should be made using the same device [67]. This parameter varies with age and sex, and should be taken into consideration when interpreting the results [68]. Vessel density also changes with retinal structural characteristics including retinal thickness and volume, and a reduced VD would correlate with thinner macular ganglion cell or inner plexiform layer [69]. Vessel density decreases in both the DCP and SCP of a patient with DR [70], as well as a diabetic patient without DR, attributing to the fact that parafoveal capillary nonperfusion in DCP may potentially be an early sign of DR [70–73].

**Fig. 2:**
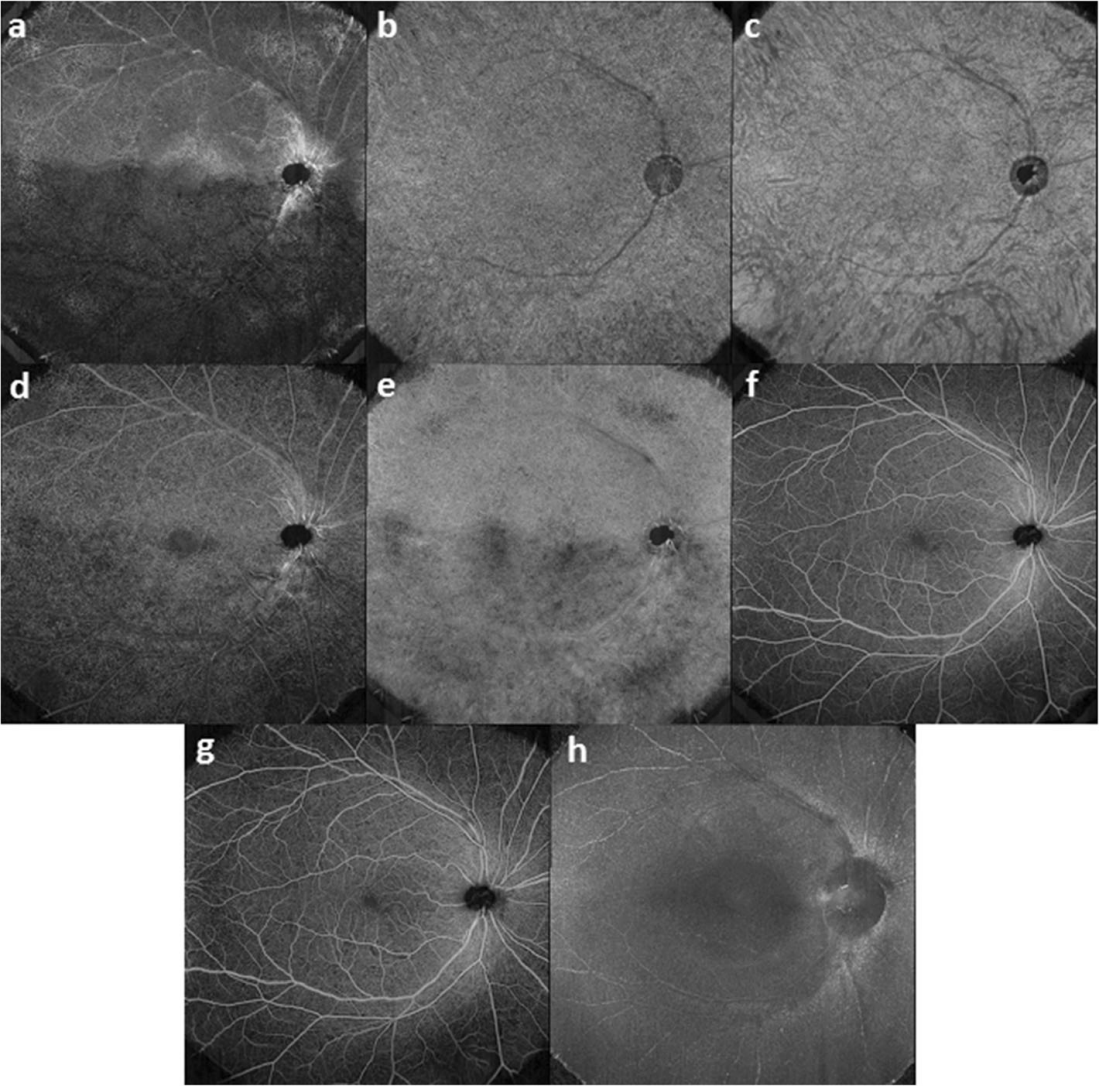
A series of montaged OCTA in patients with diabetic retinopathy (DR). This is a series of montaged OCTA images 15 mm × 15 mm taken at different segment in the right eye of a male (**a**-**h**) with DR. **a** Foveal avascular zone; **b** Choriocapillaris; **c** Choroid; **d** Deep capillary plexuses; **e** Outer-retina-choroid complex; **f** Retina; **g** Superficial capillary plexuses; **h** Vitreoretinal interface

Vessel density in DCP may predict DR severity and identify patients at risk as it is able to detect retinal vascular changes in diabetic patients without any signs of DR [71, 74]. Vascular spacing and alterations in VD in SCP, however, have found to have a stronger correlation with the severity of DR as compared to VD in DCP, PD in SCP or FAZ area [68, 70, 71]. Despite the contradictory results, VD has shown to decrease in both DCP and SCP in DR, and hence able to assist in predicting treatment outcome along with following up of patients (Fig. 2) [71, 74].

#### Inter-capillary spacing

Inter-capillary spacing can be detected by areas that are not perfused and occur much earlier than VD changes [60]. Bhanushali et al. found that large vessel spacing, especially those in the SCP, are more sensitive than VD and FAZ area in the diagnosis of DR and it reflects the severity of DR [74]. The extrafoveal avascular area may help to distinguish early NPDR from healthy eyes [75]. Schottenhamml et al. found that inter-capillary space-based algorithm is more sensitive than vascular density-based methods to calculate early capillary drop-out or non-perfusion areas [61]. As capillary non-perfusion area enlarges with progression in severity of DR, the quantitative analysis of retinal non-perfusion on OCTA may be useful for early detection and monitoring of disease in patients with diabetes and DR [76].

#### Vascular architecture and branching – vessel tortuosity and fractal dimension

Vessel tortuosity is a quantitative measure from fundus images via computer-assisted software and is defined as the integral of the curvature square along the path of vessel, normalized by the total path length [77]. Patients with diabetes have been found to have increased vessel tortuosity as compared to healthy controls and are related to mild and moderate stages of DR, suggesting that vessel tortuosity may be an early indicator of microvascular damage in the retina [78]. Vessel tortuosity may be used to distinguish moderate to severe NPDR from PDR, particularly in the SCP region. FAZ area and acircularity correlate with vessel tortuosity in 3 mm^2^ and 1.5 mm^2^ of SCP. As this parameter increases with worsening of NPDR and decreases in PDR, it may serve as a quantitative marker to monitor the progression of DR [58].

Fractal dimension is a measure of the complexity of a vasculature branching pattern [79] and is derived from applying fractal analysis to OCTA images [80]. Fractal dimension was found to be an early indicator of DR [81] and was reduced in both SCP and DCP in patients with diabetes compared to healthy controls, with a greater reduction in the DCP [82, 83].

#### Foveal avascular zone assessment

Johannesen et al. [84] conducted a systemic review on 8 studies investigating the changes in the FAZ in DR patients. Seven of these studies found that the FAZ in NPDR patients will be larger as compared with the healthy control group. Six studies on OCTA in DR found that patients with PDR have a larger FAZ as compared to the control group, and a decrease in foveal capillary perfusion in diabetics compared to controls. This increase in FAZ with the progression of DR may indicate increasing non-perfusion [85].

### Use of OCTA in macula disease in DR

**Fig. 3:**
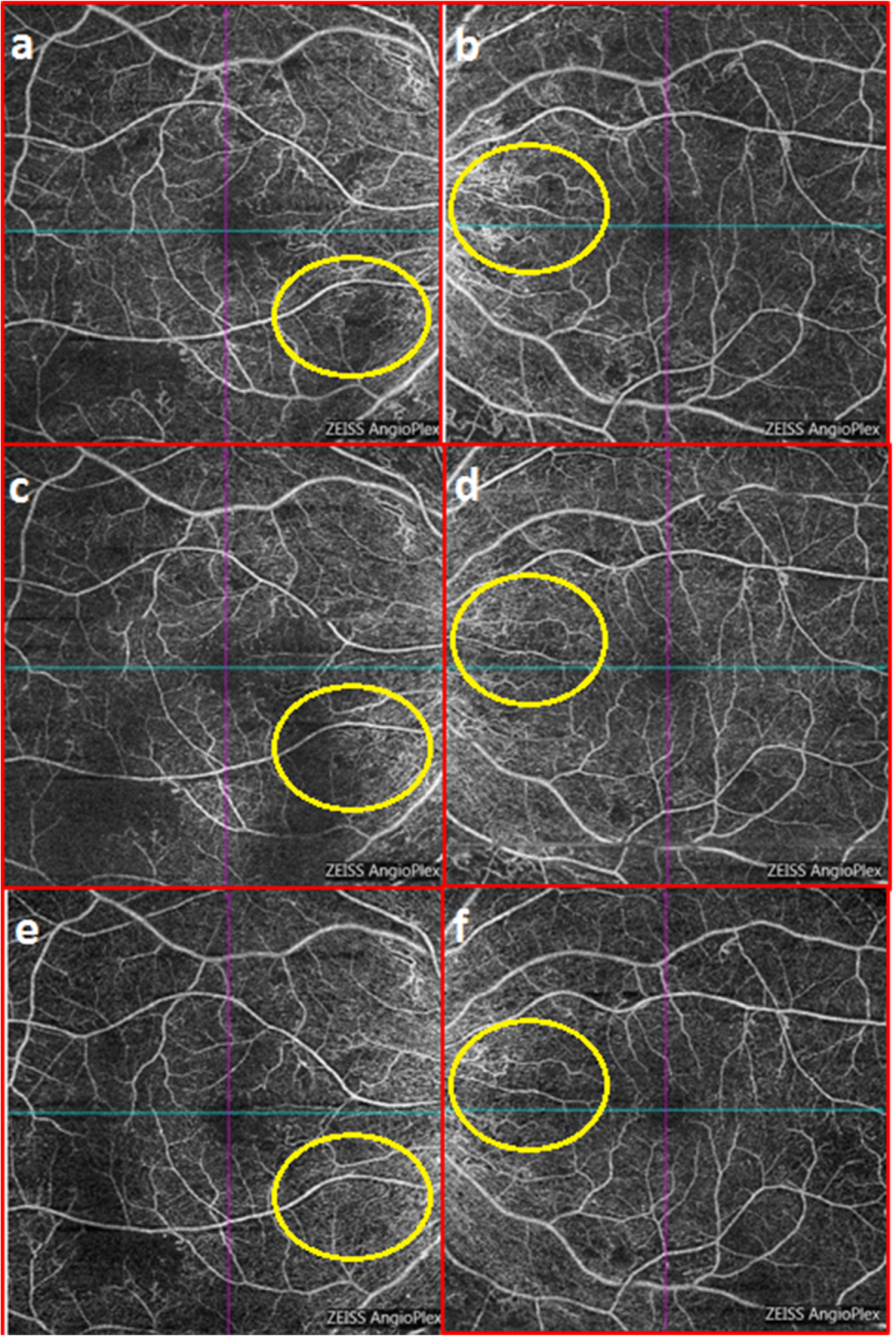
Monitoring of treatment outcome in patients with proliferative diabetic retinopathy using OCTA. This is a series of OCTA images of a 26 years old female with proliferative diabetic retinopathy taken at baseline (**a** & **b**), 1st month (**c** & **d**) and 6th month (**e** & **f**) post IVT treatment (bevacizumab). OCTA is able to detect changes - NVE regression is noted

Diabetic macular ischemia is characterized by the occlusion and loss of the macular capillary network or capillary dropout [86]. A study showed that non-perfused areas in DCP and reduced VD reflect the macular photoreceptor disruption in DMI [86, 87]. In the area of the disrupted ellipsoid zone of the photoreceptor, choroidal circulation (CC) layer had greater areas of flow void and hence alteration of CC appears to play a role in the pathogenesis of DR and DMI [88]. Wide-field OCTA images have shown that large arterioles situated in both superficial and deep layers seem to be the perfusion boundaries, which may serve as a novel anatomic factor to predict the likelihood of non-perfusion development (Fig. 3) [89] While FA is the gold standard for diagnosing DMI, OCTA may be able to do so as well [21, 86] since OCTA may provide images with higher details with respect to macular status [86] and high intergrader agreement [21]. Vascular quantitative measures of OCTA have also shown to be able to help screen and monitor DMI in patients with no clinical evidence of DR [90]. With further advancement in the technology, OCTA may serve as an alternative non-invasive method to FA to detect DMI and help predict visual prognosis.

Diabetic macular edema refers to the accumulation of fluid in the macula due to leaking blood vessels. While OCT can illustrate structural changes prominently and help in the detection of these cystic spaces [91], OCTA has low reliability in visualizing the DCP in patients with DME [92]. The accumulated fluid may interfere with imaging and segmentation capabilities of OCT as accurate identification of anatomical landmarks is needed for the complex automated process needed for correct segmentation, and incorrect segmentation may affect OCTA images [36]. DME has an inverse relationship with OCTA signal intensity [93] because the fluid weakens the reflected signal from deeper layers [94]. Spaide et al. reported that the rate of flow voiding does not match with the cystic space exactly as the vessels may be compressed by the cystic space or fluid may pool in the region of low flow rate in the DCP [95].

Regardless, Lee et al. overcame the segmentation issues by carefully adjusting the boundary between the SCP and DCP in the eyes with severe DME, and demonstrated that patients with DME exhibit significant damage to the integrity of the DCP but not the SCP [92]. It was also demonstrated that OCTA was able to assist us in quantifying macular perfusion [96] and measuring the FAZ in patients with DME [96, 97]. Using an inner segmentation of the inner retinal border and an outer segmentation of the retinal pigment epithelium, details of the macula perfusion can still be obtained in the presence of DME even though it may be difficult to differentiate between the SCP and DCP [40].

### Anterior segment optical coherence tomography angiography in diabetes mellitus

Healthy iris vasculature comprises a major arterial circle that is supplied by the anterior and long posterior ciliary arteries, and a minor arterial circle found along the border of the pupil linked by radially oriented vessels within the iris stroma. In severe stages of DR, new vessels are not confined to the retina; these can grow around the pupillary border, the root of iris and can penetrate the anterior surface of the iris in severe cases. This is known as iris neovascularization (NVI) or rubeosis which can lead to the potentially sight-threatening complication of neovascular glaucoma (NVG) [98]. It is crucial to detect NVI in its early stage as prompt treatment may prevent NVG. This complication is usually diagnosed clinically by gonioscopy and although FA may help, this is not frequently the modality of choice. A potential alternative is the use of OCTA adapted for the anterior segment [99]. While current commercially available OCTA is designed to examine the posterior segment of the eye, an adapter lens can be used to provide high-quality images of the anterior segment vasculature with a good inter-observer agreement for qualitative measurements [100]. Early studies demonstrated a method of obtaining OCTA images of the cornea and limbal vasculature with great consistency [101] and allow us to compare normal and diseased iris vessels in the detection of NVI [102].

Adapting OCTA for anterior segment does come with several downsides. Specialised anterior segment adaptive lenses have to be used [57, 101] and current software are meant for imaging the posterior segment, therefore resulting in non-parallel segmentation and artefacts due to the curvature of the cornea [103]. Anterior segment OCTA is incapable of registering scans and providing localization required for comparison of serial scans [100, 104]. In addition, motion artefacts are common in anterior scans due to a lack of motion correction software [105].

Furthermore, anterior segment OCTA is not able to visualise deeper vessels in eyes with corneal opacities, dense iris pigmentation, or vessels in thick iris tumours. It has poor detection of vessels with minimal flow since the flow of erythrocytes are slower in small calibre vessels and may be below the detection threshold. Since OCTA are optimized for the posterior segment which has mainly traversing blood flows in the vessels, anterior segment vessels with axial flow may not be detected [106].

## Conclusion

OCTA may potentially serve as a good alternative in the diagnosis and monitoring of diabetic retinopathy and maculopathy due to its non-invasiveness nature. However, the current quantitative measures developed have been more useful in research studies and their clinical implications are not yet well established. At the moment, these measures are not necessary for the diagnosis and monitoring of DR and its associated complications as there are existing methods that are clinically proven to be useful. However, with more studies being done in the near future, these quantitative OCTA measures may have a role in detecting subclinical disease. Anterior segment OCTA, especially in the imaging of the iris, may also be a useful biomarker in monitoring the progression of DR and potentially prevent severe complications.

## Abbreviations

CC: Choroidal circulation
DCP: Deep Capillary Plexuses
DME: Diabetic Macula Edema
DMI: Diabetic Macula Ischemia
DR: Diabetic retinopathy
FA: Fluorescein angiography
FAZ: Foveal avascular zone
FD: Fractal dimension
ICGA: Indocyanine green angiography
ILM: Internal limiting membrane
IRMAs: Intraretinal microvascular abnormalities
IVT: Intravitreal Therapy
MAs: Microaneurysms
NPDR: Non-proliferative diabetic retinopathy
NVE: Neovascularization Elsewhere
NVG: Neovascular glaucoma
NVI: Iris neovascularization
NVs: Neovascularizations
OCT: Optical Coherence Tomography
OCTA: Optical Coherence Tomography Angiography
PD: Vessel perfusion density
PDR: Proliferative diabetic retinopathy
SCP: Superficial Capillary Plexuses
SD: Skeleton density
VD: Vessel area density
VDI: Vessel diameter index
VLD: Vascular length density
